# Trends of Rising Research Production Among Otolaryngology Residency Applicants

**DOI:** 10.1002/oto2.170

**Published:** 2024-07-15

**Authors:** Drew H. Smith, Jad Zeitouni, Nayeon Kim Thiesse, Sarah N. Bowe

**Affiliations:** ^1^ Department of Otolaryngology–Head and Neck Surgery Texas Tech University Health Sciences Center Lubbock Texas USA; ^2^ Department of Otolaryngology–Head and Neck Surgery San Antonio Uniformed Services Health Education Consortium JBSA‐Ft Sam Houston Texas USA

**Keywords:** applicant, match, otolaryngology, publication, research, residency, selection

## Abstract

As competitiveness to obtain a residency position in the field of Otolaryngology–Head and Neck Surgery (Oto‐HNS) has continued to rise, applicants have endeavored to set themselves apart. One increasingly popular strategy is maximizing research output. Over the past 6 years, applicant metrics such as board scores and volunteer and work experiences have risen incrementally, while research production has more than doubled, from 8.4 mean number of abstracts, presentations, and publications in 2016 to 17.2 in 2022. This coincides with the exponential surge of new research fellowships among Oto‐HNS departments over a similar period, which is now up to at least 68 advertised positions. With a significant difference between the research production of matched and unmatched applicants, programs may be signaling a positive bias towards research‐heavy applicants. Whether this is intended and/or preferable should be examined more closely.

Otolaryngology–Head and Neck Surgery (Oto‐HNS) continues to be one of the most competitive specialties to enter following medical school. Electronic Residency Application Service (ERAS) and National Resident Matching Program (NRMP) data indicates that the 2023 match cycle included 526 total applicants for 373 positions in Oto‐HNS.^1^, ^2^ Securing a residency spot in Oto‐HNS is often viewed as “nearly impossible” by medical students,^3^ an accomplishment that requires years of diligent study and dedication.

Among an increasingly homogenous pool of residency applicants,^4^ there are a limited number of strategies to stand out from peers that are objectively captured in the application. The NRMP releases a detailed report of applicant data every 2 years, titled, *Charting Outcomes in the Match*.^5^ Distinct reports between MD and DO applicants started in 2016. Table 1 presents the averages of step 1 and 2 scores, and Figure 1 shows the research experiences, research output (mean number of abstracts, presentations, and publications), work experiences, and volunteer experiences among Oto‐HNS matched MD seniors from 2016 to 2022. There were incremental increases during this time period including a two‐point mean increase in Step 1 scores, a 4‐point mean increase in Step 2 scores, 2.2 more research experiences, 1 additional work experience, and 1.8 extra volunteer experiences. However, the research output more than doubled in only 6 years, from 8.4 in 2016 to 17.2 in 2022. This meaningful trend bears further investigation. Of note, DO data for Otolaryngology was excluded from the 2016 and 2018 versions of *Charting Outcomes in the Match* because “the numbers of both matched and unmatched US osteopathic seniors were smaller than 7,” and as such we chose to only present the MD data.

**Table 1:** Step Scores of Otolaryngology‐Matched MD Seniors

Year	Step 1	Step 2
*2022*	250	257
*2020*	248	256
*2018*	248	254
*2016*	248	253

**Figure 1:**
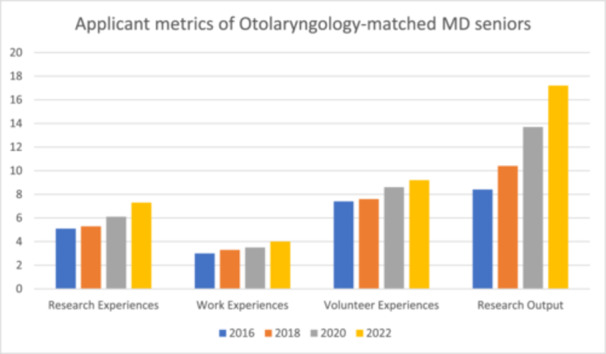
Applicant metrics of Otolaryngology‐matched MD seniors. Research output represents the mean number of abstracts, presentations, and publications.

Several theories to explain the increase in research output are plausible. One explanation is the rise of student research positions among otolaryngology departments. These research fellowships typically last 9 months to 1 year and are often undertaken between the third and fourth years of medical school, although fellowship programs also frequently hire graduated, unmatched Oto‐HNS applicants. It is difficult to quantify the number of research fellowships that were available in the past, but in 2018 there were online postings in the forum and chat sections of the applicant‐popular *Otomatch* website for 3 available fellowships.^6^ Recently, these fellowships have become much more commonplace among Oto‐HNS departments. Examining otolaryngology academic department websites, we were able to identify 29 fellowships offering a total of 68 positions as of November 1, 2023 (Table 2). Nearly all the positions are compensated, with a range of $25,000 to $30,000. While it is likely that more positions existed in 2018 than we were able to pinpoint, it is also expected that more exist now than are advertised (the lead author completed a research fellowship in 2020 that was personally arranged and never promoted online). Nevertheless, the rise in number of fellowships is certainly an evolving trend and likely contributing to the increased research output among applicants. It is unclear whether the current research fellowship upsurge is an effect of the COVID pandemic, but it is conceivable that this era's increased awareness of remote work possibilities and dependence on existing research databases led departments to recognize the opportunities available to bolster both its own and its medical students' research output through dedicated research positions.

**Table 2:** Research Fellowships Advertised by Otolaryngology Departments in the United States

Department	# of positions	Funded
University of Arkansas for Medical Sciences	1	Yes
University of California Los Angeles	2	Yes
University of California San Diego	1	Yes
University of California San Francisco	1	No
Stanford University	1	Yes
MedStar Health/Georgetown University	1	No
University of Florida	3	Yes
University of Miami/Jackson Health	2	Yes
Rush University	1	Yes
University of Iowa	1	Yes
University of Kansas	5	Yes
Mayo Clinic (Rochester)	2	Yes
Washington University	1	Yes
Icahn School of Medicine at Mount Sinai	4	Yes
New York University	1	Yes
Duke University	2	Yes
Case Western Reserve University/University Hospitals	2	Yes
Penn State	1	Yes
Sidney Kimmel Medical College at Thomas Jefferson University	9	Yes
University of Pennsylvania	1	Yes
University of Pittsburgh	1	Yes
Medical University of South Carolina	12	Yes
University of Tennessee	4	Yes
Vanderbilt University	3	Yes
Methodist Hospital (Houston)	1	Yes
University of Utah	1	Yes
University of Virginia	2	Yes
Virginia Commonwealth University	1	Yes
University of Washington	1	Yes

Understanding whether applicant research production has a dominant influence on program directors is imperative to medical students considering a research year. Among responding otolaryngology program directors in the 2021 program director survey produced by the NRMP, only 78.6% endorsed “involvement and interest in research” as a factor when considering whom to interview.^7^ While there are nearly innumerable additional influences that take an applicant from the interview stage to the matched stage, the gap in research output between matched and unmatched MD applicants in 2022 was notable at 17.2 and 11, respectively.^5^ Program directors may have a degree of bias when it comes to favoring applicants that produce more research.

It is also possible that there exists an unknown amount of conflation in current research metrics compared to what applicants actually produce. A 2018 paper investigated the rates of misrepresentation of scholarly work and found that 6.2% of otolaryngology applicants had inflated their research output, most commonly by elevating themselves to a higher authorship status or listing nonpeer‐reviewed items as peer‐reviewed.^8^ The authors characterized this rate as low, but it is conceivable that increasing competitiveness has led applicants to further inflate their research metrics in more recent years. Additional study is warranted to evaluate this possibility.

Applicants may feel that research is an area where placing additional time and resources can translate into a quantifiable edge. This is only likely to increase as Step 1 transitions to a pass/fail mark for future match cycles. A 2015 commentary in this journal accurately forecasted the rise in both medical students participating in research fellowships and applicant research production.^3^ They warned that the trend of taking an additional research year during medical school to gain a competitive advantage could put applicants who cannot do so due to financial constraints at a disadvantage. While many of the new research positions are paid, the funds typically do not exceed many students' monthly rent. Many of these positions are located in metropolitan cities with high costs of living which likely necessitates that students rely on additional means, including extra loans, to fully fund their research year. While learning the process of scientific investigation is certainly a vital passage of medical training, the utility of research production as a central metric to the application process and a predictor of resident success is questionable. Further discussion should be held among program directors and other graduate medical education leaders concerning these trends. Additional criteria or traits that can be employed to better differentiate between applicants using holistic review should be identified.^9^

## Author Contributions

**Drew H. Smith**, concept, design, data acquisition, writing, editing, final approval; **Jad Zeitouni**, data acquisition, writing, editing, final approval; **Nayeon Kim Thiesse**, data acquisition, editing, final approval; **Sarah N. Bowe**, design, editing, final approval.

## Disclosure

### Competing interests

None.

### Funding source

None.
